# Double contrast-enhanced ultrasonography of a small intestinal neuroendocrine tumor: a case report of a recommendable imaging modality

**DOI:** 10.1093/pcmedi/pbaa011

**Published:** 2020-04-17

**Authors:** Jie-ying Zhao, Hua Zhuang, Yuan Luo, Ming-gang Su, Mo-li Xiong, Yu-ting Wu

**Affiliations:** Department of Ultrasound, West China Hospital of Sichuan University, 37#Guo Xue Xiang, Chengdu, Sichuan 610041, China; Department of Ultrasound, West China Hospital of Sichuan University, 37#Guo Xue Xiang, Chengdu, Sichuan 610041, China; Department of Ultrasound, West China Hospital of Sichuan University, 37#Guo Xue Xiang, Chengdu, Sichuan 610041, China; Department of Nuclear Medicine Imaging, West China Hospital of Sichuan University, 37#Guo Xue Xiang, Chengdu, Sichuan 610041, China; Department of Pathology, West China Hospital of Sichuan University, 37#Guo Xue Xiang, Chengdu, Sichuan 610041, China; Department of Ultrasound, West China Hospital of Sichuan University, 37#Guo Xue Xiang, Chengdu, Sichuan 610041, China

**Keywords:** double contrast-enhanced ultrasonography, neuroendocrine tumor, small intestinal tumor, carcinoid

## Abstract

A 57-year-old male presenting with spontaneously relieved abdominal cramp and distension was admitted to the West China Hospital. The diagnosis remained unclear after colonoscopy and computed tomography. Double contrast-enhanced ultrasonography was then performed and a neoplasm in the small intestine was suspected, supported by a thin-section computed tomography and positron emission tomography/computed tomography. This was confirmed pathologically after surgery to be a small intestinal G1 neuroendocrine tumor. Surgery was performed to remove approximately 25 cm of small bowel and a 3-cm solid mass located in the mesentery. The patient had a complete recovery and was tumor-free at the final follow-up. Small intestinal tumors including neuroendocrine tumors have always posed a diagnostic challenge. This case indicated that double contrast-enhanced ultrasonography is feasible in detection of small intestinal neuroendocrine tumors, and it may be an advisable approach assisting diagnosis of small intestinal tumors.

## Introduction

Compared to large intestinal tumors, small intestinal tumors (SITs) are uncommon, and present a diagnostic challenge because of vague symptoms, nonspecific radiologic appearance, slow growth, and long disease progression. Existing imaging examinations, including conventional ultrasound, do not provide satisfactory diagnostic sensitivity.^1^ Patients with small intestinal malignant tumors have a poorer prognosis than those with colorectal malignant tumors.^1,2^

Neuroendocrine tumors (NETs) of the small intestine are even rarer.^3,4^ The clinical manifestations of NETs usually occur at late phase of the disease, giving rise to intestinal obstruction, thus use of endoscopy or capsule endoscopy is limited.^5^ It is difficult to choose an optimal diagnostic imaging modality for small intestinal NETs. Accurate imaging of NETs is critical to management decisions, which should be tailored to answer relevant clinical questions according to ENETS Consensus Guidelines for the Standards of Care in Neuroendocrine Tumors.^1^

Double contrast-enhanced ultrasonography (DCEUS) is a newly developed approach, which combines use of oral and intravenous contrast agents to visualize vascular-rich neoplasms in the filled lumen without gas impairment.^6^ This approach has been verified as capable of improving the ability of ultrasound in detection of gastric, periampullary and colorectal lesions.^6-11^ In the case reported here, a small intestinal NET was detected by DCEUS. Being non-invasive, repeatable, favorable at depicting small bowel masses, and providing real-time information,^3,12^ DCEUS may offer a useful supplement in detection and diagnosis of SITs.

## Case report

A 57-year-old male was admitted to our hospital with ‘suspicious intestinal obstruction’, presenting with spontaneously relieved abdominal cramp and distention for 6 months without flushing, secretory diarrhea, tachycardia, or bloody stools. The local hospital failed to provide a diagnosis. The vital signs of the patient at the time of admission were normal. No positive sign was found, except tenderness in the right lower quadrant abdomen. Laboratory examinations including NET-related hormonal tests were normal (e.g. serotonin, histamine, dopamine, and hydroxytryptophan), except for a slight decrease in T cells (CD3: 54%, CD4: 31.8%, CD8: 18.2%) and total protein (63.3 g/l). The patient had a clear previous medical history, and his family history was negative.

The colonoscopy revealed nothing of importance (only mixed hemorrhoids). Contrast-enhanced computed tomography (CT) of the abdomen showed only a segmentally thickened small bowel wall in the ileocecal region. A diagnosis of inflammatory bowel disease was suspected, which was not supported by endoscopy. Given the uncertainty of intestinal obstruction, capsule endoscopy and enteroscopy were not performed.

**Figure 1.**
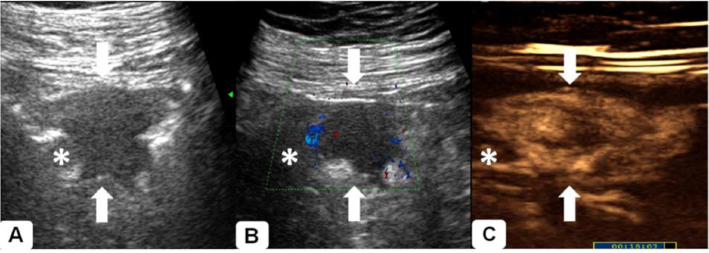
DCEUS of a small intestinal NET. **(A)** A hypoechoic mass (arrow) was revealed in the periumbilical area, partly obstructing the lumen of the small intestine. **(B)** Dot-like blood signals were found inside the mass (arrow). **(C)** The mass (arrow) after DCEUS, and stratification of the bowel wall was partly interrupted. (Asterisk: the proximal bowel lumen filled with homogenous echoic and non-enhancing oral contrast agent.)

The result of conventional ultrasound of abdomen was normal. DCEUS was performed as follows. Firstly, 500 ml of intraluminal contrast (commercially available Tianxia brand, East Asia Institute of Gastrointestinal Ultrasound, Huzhou, China) was taken orally, 4 hours after intestinal wash (with 1500 ml sodium phosphate liquid). Secondly, after 30 minutes, 2.4 ml of Sonovue (Bracco, Milan, Italy) was injected intravenously to conduct DCEUS. A hypoechoic mass 30 mm × 16 mm in size with irregular shape and dot-like blood signals inside was revealed in the periumbilical area with oral contrast, partly obstructing the lumen of the small intestine, yet without prominent proximal intestinal dilation (Fig. 1A and B). The mass was highly vascular as it enhanced earlier than normal surrounding tissue after intravenous contrast agent injection (Fig. 1C). The mass was heterogeneously hyper-enhanced with hypo-enhanced area inside, and stratification of the bowel wall was partly interrupted. A neoplasm of the small intestine was suspected according to ultrasonic findings. No discomfort was declared during the procedure.

**Figure 2.**
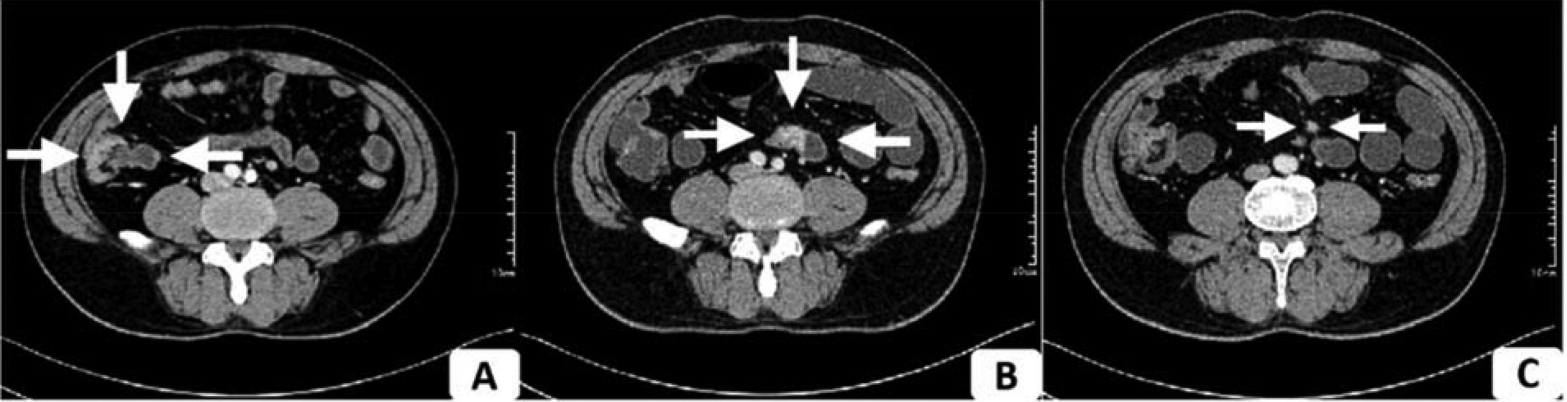
Abdominal thin-section contrast-enhanced CT. **(A)** Ileocecal intestine thickening (arrow). **(B)** Jejuno-ileum wall thickening and heterogeneous enhancement, with a 2.1 cm heterogeneously enhanced mass (arrow) obstructing the lumen. **(C)** Surrounding enlarged lymph node (arrow) and mesenteric kinks.

**Figure 3.**
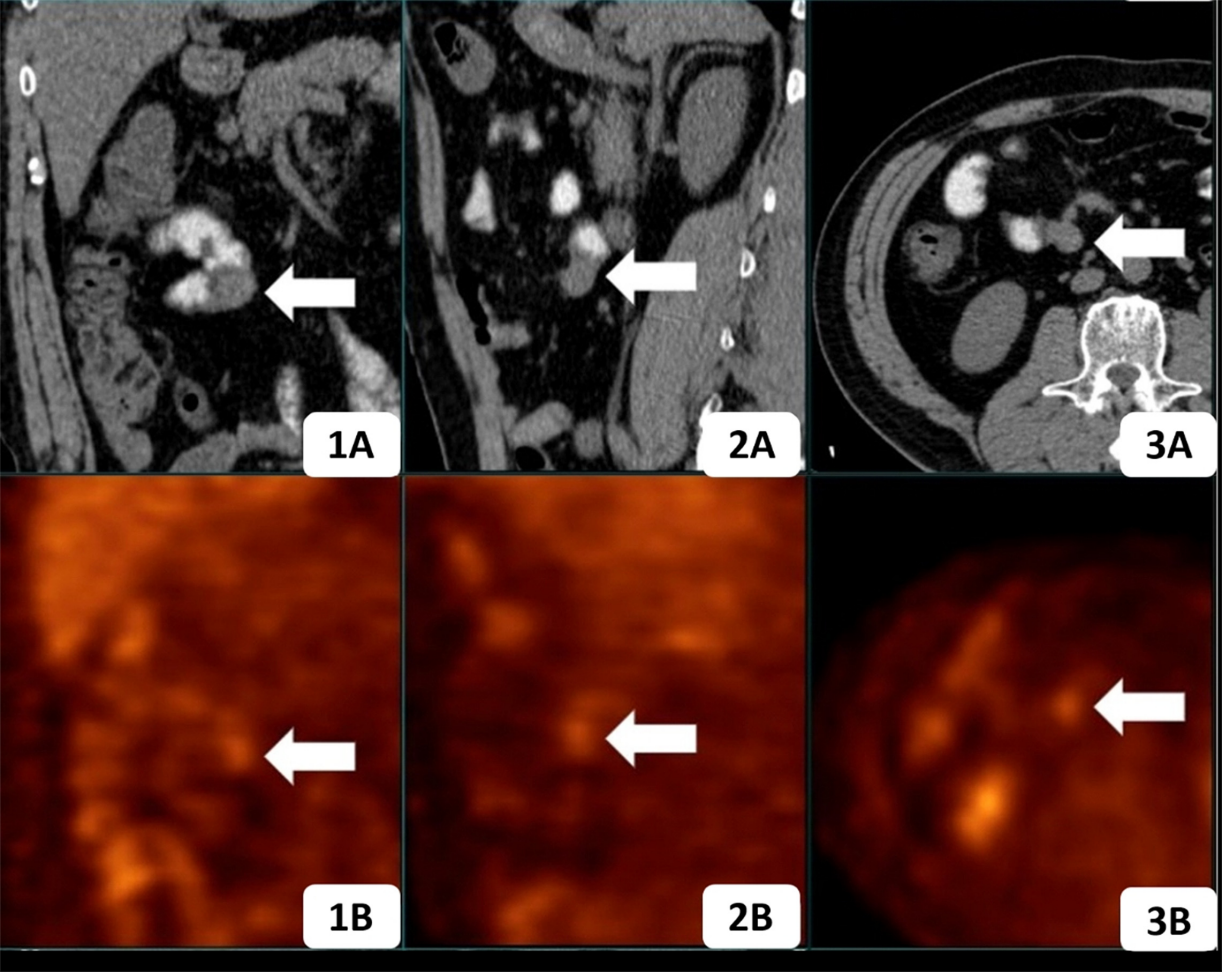
Coronal (1A), sagittal (2A), and axial (3A) abdominal CT with oral contrast showed an obscure small intestinal mass (arrow), with a diameter of 30 mm. The corresponding PET images (1B–3B) revealed slightly accumulated 18F-FDG (arrow), with a maximum standardized uptake value of 2.35.

To verify the ultrasonic findings, a thin-sectional CT was recommended. Opposite to the result of the former contrast-enhanced CT scan, a heterogeneously enhanced mass with a diameter of 2.1 cm was discovered, obstructing the lumen at the junction of the jejuno-ileum, with no distant metastases in the upper abdomen (Fig. 2). Following (PET)-CT showed a very obscure lesion with an almost imperceptible elevation of standard uptake value (SUV) of 2.35 in the right lower abdominal quadrant, suggesting either a benign or low-invasive lesion (Fig. 3).

**Figure 4.**
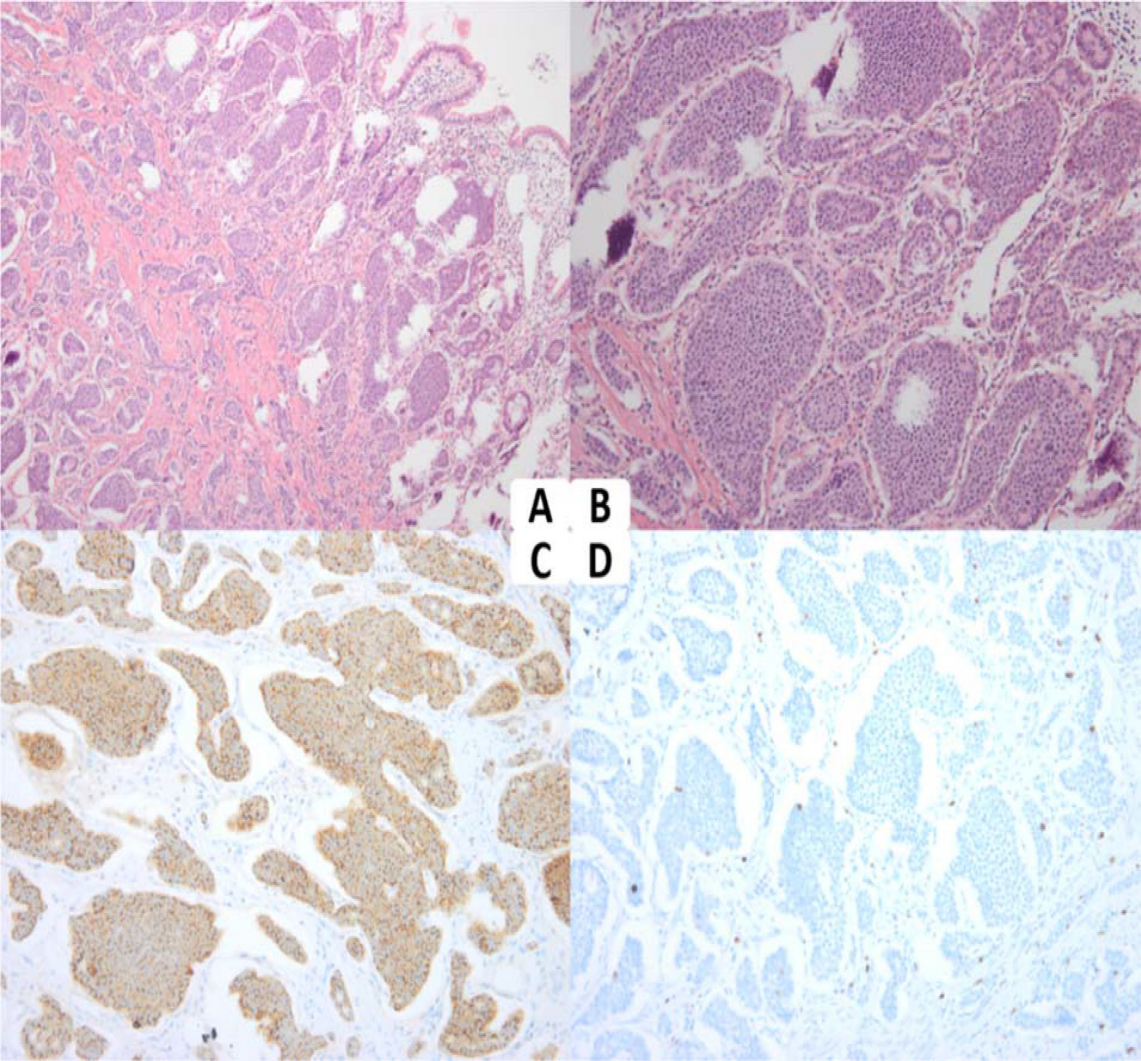
Histopathological results. **(A)** Hematoxylin and eosin staining (100x) showing the nesting pattern growth. **(B)** Hematoxylin and eosin staining (200x) showed that the tumor was composed of small relatively uniform cells with centrally located nuclei and acidophilic or amphiphilic, fine granular cytoplasm. **(C)** Immunohistochemistry showed that chromogranin A was positive. **(D)** The proliferation index of Ki-67 was 2%.

Surgery was performed to remove approximately 25 cm of small bowel, including a 3-cm hard mass located in the mesentery of the intestine, as well as the surrounding fibrotic mesentery. Histopathologically, the final diagnosis was a G1 NET (Fig. 4) of the small intestine, which had invaded the subserosa and two out of three adjacent lymph nodes. The patient had a complete recovery after surgery and was tumor-free at the final follow-up.

## Discussion

SITs have always posed a diagnostic challenge because of their rarity, deep abdominal location, morphological tortuosity, and vague symptoms such as abdominal pain or discomfort.^3,4^ In case of SITs, fasting and water drinking are necessary for nearly all the imaging modalities. Previously, CT and MRI were basic modalities to detect small bowel disease;^1^ however, because of the radiation involved, CT is not advisable for frequent screening. Despite its good soft tissue resolution, MRI is a time-consuming process, producing artifacts caused by small bowel motion. At times, static images show the flaws of CT and MRI,^3,13,14^ which is rarely the case for ultrasound because it is a real-time process. PET-CT provides a thorough view of the body, but is not suggested for routine detection because of its low space resolution.^14^ Capsule endoscopy (CE) and enteroscopy are recommended for patients with obscure GI bleeding, but are restrictive for SITs because of the risk of capsule retention as well as miss rate through quick transit.^2,5,15^

NETs are secondary malignancies of the small intestine following adenocarcinoma, originating from secretory cells of the diffuse neuroendocrine system, with an estimated incidence of 5/100 000.^16,17^ Their frequency of occurrence correlates with the site-density of neuroendocrine cells and almost 60% of carcinoid tumors arise from the intestine, with the last 60 cm of the terminal ileum being the most common site.^17^ NETs are slow-growing and most are found incidentally. Some NETs are hormonally active so hormonal examinations may be abnormal (e.g. serotonin, histamine, dopamine, and hydroxytryptophan). Most typical symptoms (e.g. flushing, secretory diarrhea, tachycardia, etc.^16,18^) occur in the late phase of the disease when metastases happen.^19,20^ In addition, NETs possess similar radiologic appearance to other SITs or retractile mesenteritis, which makes their diagnosis more difficult.^19,21^ Pathology is the gold standard of diagnosis. Surgery is the only curative treatment for NETs and adjuvant chemotherapy can achieve prolonged progression-free survival and symptom relief.^22,23^

Conventional ultrasound is recommended for diagnosis of liver NETs and biopsy guidance for metastases.^1^ Intraoperative ultrasound also facilitates lesion detection in the pancreas and liver, according to the ENETS Consensus Guidelines in 2017.^1^ However, this is not well accepted in gastrointestinal NETs because of the impediments of the intraluminal gas and feces.^24^

Some scholars have suggested that DCEUS is helpful for detecting and characterizing masses in and around the bowel loops.^6,8^ With a combination of oral and intravenous contrast agents, DCEUS provides a good acoustic window for evaluation of gastrointestinal tumors with a better contrast to surrounding intestinal mural structures. Oral contrast agent fills and so visualizes the lumen, while intravenous contrast agent delineates the tumor extent and vascularity, which is crucial for lesion characterization.^25^ NETs are often highly vascular after contrast agent injection and their wash-out correlates with their malignant potential.^20^ If the patient is not obese, we can even judge the infiltration and stage status of tumors according to the disruption of wall integrity.

There are several reports on successful use of DCEUS in gastric cancer and colorectal adenocarcinoma, both at the initial diagnosis and the tumor stage assessment. Yan *et al*.^9^ verified that the overall accuracy of DCEUS in determining the gross appearance of gastric carcinoma was higher than that of multi-detector computed tomography (84.9% vs. 79.9%, *P* < 0.001). Zheng *et al*.^7^ compared the accuracy of endoscopic ultrasound with DCEUS in staging of gastric malignancies. Their results indicated that the overall accuracy of DCEUS for tumor (T) staging was 77.2%. DCEUS (78.4%) was superior to endoscopic ultrasound (57.4%) in lymph node detection while a slight advantage was presented only for a tumor depth of T3. Zhuang *et al*.^8^ used DCEUS to quantify microcirculation of colorectal adenocarcinomas and the area under the time–intensity curve within the tumors was significantly different in the subgroups of different T stage. They also studied colorectal tumor angiogenesis and the biological behavior by DCEUS.^6^ Lu *et al*.^10^ used DCEUS to differentiate rectal adenocarcinomas, adenomas, and inflammatory masses. The morphologic characteristics and perfusion parameters varied among different lesions, therefore DCEUS may help to differentiate benign and malignant rectal lesions. Nevertheless, there are few reports on use of DCEUS in SITs. Zhang *et al*.^11^ indicated that the sensitivity and specificity of DCEUS were not significantly different from MRI in periampullary cancer, and both DCEUS and MRI were superior to conventional ultrasound.

In our case, except for uncertain bowel obstruction, neither NET-related symptoms nor distant metastases were found. The diagnosis was initially confused. At the beginning, the tumor was discovered neither by endoscopy nor by CT. Oral contrast agents helped visualize the mass and CEUS differentiated the tumor from abscess. Thus DCEUS was the first modality to take small bowel neoplasm into consideration. If capsule endoscopy was performed, the risk of capsule retention may have failed the process. The tumor may have been overlooked if DCEUS had not been performed.

In conclusion, it is difficult to detect small intestinal NETs because of obscure clinical symptoms at the early stage. DCEUS is non-invasive, repeatable, and radiation-free, with the ability to clearly depict vascularity of masses in filled lumen. We verified that DCEUS is feasible in detecting small intestinal NET and we infer that it may be an advisable approach assisting diagnosis of SITs. As diagnosis of small intestinal SITs remains a challenge for conventional imaging modalities, the clinical value of DCEUS in SITs should be further validated with large cohorts.
